# Intensified Pulse Rotations Buildup Pea Rhizosphere Pathogens in Cereal and Pulse Based Cropping Systems

**DOI:** 10.3389/fmicb.2018.01909

**Published:** 2018-08-23

**Authors:** Yining Niu, Luke D. Bainard, William E. May, Zakir Hossain, Chantal Hamel, Yantai Gan

**Affiliations:** ^1^Swift Current Research and Development Centre, Agriculture and Agri-Food Canada, Swift Current, SK, Canada; ^2^State Key Laboratory of Aridland Crop Biology, Gansu Agricultural University, Lanzhou, China; ^3^Indian Head Research Farm, Agriculture and Agri-Food Canada, Indian Head, SK, Canada; ^4^Quebec Research and Development Centre, Agriculture and Agri-Food Canada, Quebec City, QC, Canada

**Keywords:** cropping system, field pea, high throughput sequencing, pathotrophs, rhizosphere fungal diversity, soil physicochemical properties, *Olpidium virulentus*

## Abstract

The association of plants and microbial communities is crucial for crop production, and host plants influence the composition of rhizosphere microbiomes. Pulse crops play an important role in the development of sustainable cropping systems, and producers in the Canadian prairies often increase the frequency of pulses in their cropping systems. In this study, we determined the shifts in the fungal community of pea (*Pisum sativum* L.) rhizosphere, as influenced by the frequency of pulses in rotation, using high throughput sequencing. Six cropping systems containing pea (P), lentil (*Lens culinaris* Medik., L), hybrid canola (*Brassica napus* L., C), wheat (*Triticum aestivum* L., W), and oat (*Avena sativa* L., O) in different intensities were tested. The fungal communities were assessed at the flowering stage in the fourth and fifth year of the 4-year rotations. Cropping system had a significant impact on the composition of the rhizosphere fungal community, and the effect of crop rotation sequence was greater and explained more of the variation than the effect of previous crops. The rotation with consecutive pulses (WPLP) decreased fungal evenness and increased the proportion of pathotrophs. *Fusarium* was a dominant and ubiquitous pathotrophic genus. *Olpidium virulentus, Botrytis cinerea, Fusarium solani, F. graminearum*, and *Alternaria eichhorniae* were generally more abundant in pulse intensive rotations (WPLP, WLOP, and WPOP), the exception being *F. solani* which was not promoted by lentil. Reads of *O. virulentus* and *B. cinerea* were most abundant in pea preceded by lentil followed by the reads of *Mortierella elongata* in pea preceded by wheat. Pea consistently had higher grain yield when grown in diversified rotations including wheat, canola/lentil, and oat than rotations with two repeated crops (canola or pea). Cropping system affected the soil physicochemical properties, and soil pH was the main driver of fungal community shift. No evidence of beneficial microorganisms involvement in plant productivity was observed, but the high abundance of pathotrophs in pulse intensified rotations suggests the possibility of pathogen buildup in the soil with increasing pulse frequency. Diversifying rotation sequences minimized disease risk and increased pea production, in this study. Careful selection of plant species appears as a strategy for the management of rhizosphere fungal communities and the maintenance of crop production system’s health.

## Introduction

Agroecosystem productivity is often restricted by water availability, and the challenge is serious in many arid and semiarid regions of the world (Borontov et al., 2005; Rasouli et al., 2014; Gan et al., 2015). Summer fallow has historically been an important farming practice to mitigate the impact of moisture deficit in dry areas, including the Canadian prairies (Grant et al., 2002). However, summer fallowing practices have serious environmental consequences. The introduction of pulse crops, such as field pea, lentil, and chickpea (*Cicer arietinum* L.) into the traditional cereal-fallow-based agricultural systems has increased agricultural productivity while decreasing environmental impacts (MacWilliam et al., 2014; Gan et al., 2015; Gupta et al., 2015). Pulse crops have also changed soil biology and biological processes (Lupwayi and Kennedy, 2007). In particular, cereal crops producing good grain yield are highly N use efficient when they follow N_2_-fixing legumes in rotation largely because lesser amounts of fertilizer are required (Luis et al., 2014). The introduction of pulses in rotation influences N_2_-fixing bacteria, and may well influence the overall microbial environment of crops. On the northern Great Plains of North America, there is a trend of increasing the frequency of pulses in cropping systems. However, information on how the frequency of pulse crops in cropping systems impacts the soil microbial community is lacking.

Soil microbial communities are the most abundant reservoir of known biological diversity (Berendsen et al., 2012). Plants live in close association with the soil microbes and are able to shape microbial communities. The root microbiome of plants grown in the same soil differ between plant species (Garbeva et al., 2008; Berg and Smalla, 2009; Berendsen et al., 2012), and some plant species can forge similar microbial communities in different soils (Miethling et al., 2000). Plants exert such influence on the composition of rhizosphere microbiome by secreting compounds that selectively stimulate or repress members of the community (Doornbos et al., 2012). Soil fungi are an important component of the soil microbiome and contribute to key processes in soil (Joergensen and Wichern, 2008). Fungal communities can increase plant resilience to abiotic stress, cause diseases as well as reduce the incidence of diseases, and provide the ecological services of nutrient cycling (Bridge and Spooner, 2001; Lupwayi and Kennedy, 2007; Penton et al., 2014). The composition of the fungal community of crop rhizosphere is an important determinant of crop success (Appuhn and Joergensen, 2006; Berendsen et al., 2012). Variations in the composition of rhizosphere fungal communities are partly attributable to plant host effects (Bonito et al., 2014).

Pea is one of the pulse crops most widely used in the northern Great Plains. Inclusion of pea in cropping systems enhances soil water conservation, increases soil N level, and system productivity (Gan et al., 2015). The improved performance of crops is partly due to the stimulation of beneficial soil microorganisms, including plant growth-promoting microorganisms and biocontrol agents (Lupwayi and Kennedy, 2007; Vujanovic et al., 2012; Yang et al., 2013). However, frequent pea crops in cropping systems had negative effects, including decreased net income (Nielsen and Vigil, 2017), reduced soil fungal diversity, increased proportion of fungal pathotrophs (Bainard et al., 2017), depressed soil organic carbon level, and dehydrogenase, phosphatase, and urease activity (Nayyar et al., 2009). In the present study, we focus on the relationship between the composition of the fungal community of the pea rhizosphere and the frequency of pea and pea preceded crops in pulse and cereal based cropping systems.

Producers in the Canadian prairies consider pulses as economically beneficial crops and often increase the frequency of pulses in their cropping systems. However, how increased pulse frequency in cropping systems may affect soil biological quality is largely unknown. In this study, we evaluated the impact of cereal, oilseed and pulse-base cropping systems on the fungal community of the pea rhizosphere. The specific objectives of this research were to (1) determine the effect of cropping system on the composition and diversity of the fungal community of pea rhizosphere, (2) relate the changes in soil properties induced by cropping systems to the variation in fungal community composition, and (3) determine the impact of cropping systems or fungal communities on the agronomic performance of pea. We hypothesize that the pea rhizosphere fungal community is significantly affected by pea crop frequency and sequence under different cropping systems, and in turn affects pea growth and productivity.

## Materials and Methods

### Site Description and Experimental Design

The field experiment was conducted from 2012 to 2016 at the Agriculture and Agri-Food Canada Research Farm in Indian Head (50°32′N, 103°40′W), Saskatchewan. The soil was a Redo Black Chernozem (Udic Boroll) with saturated paste pH of 7.1 in the 0–15 cm depth, and a heavy clay texture, as it contains 15.0% sand, 32.5% silt, and 52.5% clay.

The experiment had six cropping systems each with four phases. All rotation phases were present every year. Treatments were laid in a randomized complete block design with four repetitions, in 96 plots. Plot sizes were 4 m × 12 m. In the 4-year rotation cycle, pea (P), lentil (L), hybrid canola (C), spring wheat (W), and oat (O) were seeded in different intensities to constitute six cropping systems: CWCP, WCLP, WCOP, WPCP, WPLP, and WPOP (**Supplementary Table S1**). The first rotation cycle started in spring 2012 and was completed in fall 2015, and second cycle commenced in 2016.

Crops were seeded in early May each year and managed according to recommended agronomic practices. Wheat and pulse seeds were treated with Vitaflo 280 (containing carbathiin 15.59% and thiram 13.25%) and Apron Maxx (containing fludioxonil 0.73% and metalaxyl-M and S-isomer 1.10%), respectively, at a rate of 3.3 ml kg^-1^ of seed. Pulse seeds were inoculated with commercial granular *Rhizobium* inoculants following manufacturer’s recommendation before seeding. The field target N rate (soil residue N plus fertilizer N) was 110 kg ha^-1^ for wheat, 120 kg ha^-1^ for canola, and 80 kg ha^-1^ for oat in the top 0–60 cm soil layer (**Supplementary Table S2**). No N-fertilizer was applied to the pulse crops (pea and lentil). The P, K, and S fertilizers were applied to the crops based on soil test recommendation made on samples taken in fall. Glyphosate, Roundup WeatherMax and Bonanza 10G were applied separately or combined when needed for weed control.

### Weather Conditions

The average monthly temperature from pea sowing to flowering stage (May to July) was 15.8°C in 2016 and 14.8°C in 2015, which is similar to long term average (14.9°C) (**Supplementary Table S3**). The cumulative precipitation in 2015 was 148.5 mm, 14.1% lower than the long-term average (190.2 mm). In 2016, the precipitation increased to 232.8 mm, which is 22.4% higher than the long-term average. The precipitation during the crop-growing period (May to August) was 207.3 and 254.7 mm in 2015 and 2016, respectively, while the average temperature was 15.3 and 16.1°C (**Supplementary Table S3**).

### Soil Sampling

Rhizosphere and bulked soil samples from pea plots were collected at flowering on 15 July 2015 and 18 July 2016. For the rhizosphere soil samples, three points (4–5 plants per point) were randomly selected in a plot and plant roots were excavated. Shoots were detached with scissors and roots with adhering soil were placed in labeled Ziploc bags, and immediately stored in a cooler. In the laboratory, rhizosphere soil was brushed off the root surface and passed through a 2 mm sieve to remove rocks and roots, then immediately stored at -20°C.

Bulked soil samples from the crop row were also collected at the same time as the rhizosphere soil and the soil physicochemical properties of the bulk soil were determined. In each plot, six soil cores (2 cm diameter and 15 cm deep) were collected from three selected points, homogenized, and combined to form a composite sample. After soil sample was passed through 2 mm sieve, a subsample was used for soil moisture measurement, and the remainder was air dried to determine soil pH, electrical conductivity, and soil nutrient analysis.

### Molecular Analyses

Total DNA was extracted from approximately 0.25 g of rhizosphere soil using the PowerSoil DNA Isolation Kit (MO BIO Laboratories, Inc., Carlsbad, CA, United States) following the manufacturer’s instructions. The DNA concentration was determined using Qubit 3.0 Fluorometer (Thermo Fisher Scientific). The DNA was transferred to 96-well plates and shipped to Genome Quebec for high throughput sequencing (Illumina MiSeq PE250). The universal fungal primer pair ITS1F (Gardes and Bruns, 1993) and 58A2R (Martin and Rygiewicz, 2005) was used to amplify the ITS1 region of the rhizosphere fungal community.

### Data Analysis

Illumina paired sequences were merged and trimmed to remove primers following the procedure of UPARSE pipeline (Edgar, 2013) in USEARCH (Edgar, 2010). Sequences were dereplicated and all singletons were excluded from further analysis. Sequences were then clustered into operational taxonomic units (OTUs) based on 97% similarity, and OTUs were mapped back to the samples using *usearch_global* command. The RDP classifier (Wang et al., 2007) was used to assign taxonomy to the OTUs (Dufrene and Legendre, 1997) using the UNITE fungal ITS database (Abarenkov et al., 2010). FUNGuild was used to parse the fungal community datasets by functional ecological guild (Nguyen et al., 2016).

Samples were randomly resampled to the lowest number of reads (5370) prior to statistical analysis to allow comparison of all samples on an equal basis using the *rrarefy* command of the *vegan* package (Oksanen et al., 2016) in R. The Chao1 richness (Chao, 1987; Chiu et al., 2014) estimator was calculated using the *estimateR* function and the Pielou’s evenness indices determined using the *diversity* function of the *vegan* package.

For cropping system effect determination, we analyzed the rotation sequence effect, this include the effect of the crop sequence and pulse crop intensity in the rotations. Under different rotation sequences, only pea in the same phase preceded by canola, lentil, and oat were considered in the ANOVA model. Pea preceded crop was analyzed to determine the pulse crop sequence effect. The effect of previous crops (wheat, canola, lentil, and oat) on the fungal community of pea rhizosphere and pea grain yield, was analyzed independently among the rotations WPCP, WPLP, and WPOP, which included two pea crops. Similar crops in the rotations were pooled together (i.e., wheat = Wpcp, Wplp, Wpop; canola = wpCp; lentil = wpLp; and oat = wpOp) in the analysis of previous crop.

ANOVA were conducted using the *lmer* function of package *lme4* (Bates et al., 2015) in R to test the effects of year, rotation sequence or previous crop (fixed factors), and block (random factor) on soil chemical properties, rhizosphere fungal diversity indices, relative abundance of OTU reads, fungal functional guilds, and pea grain yield. Preliminary analyses indicated that there was significant (*P* < 0.05) variation between year 2015 and 2016, therefore the effect of rotation sequence or previous crop were analyzed by year. The normal distribution and the homoscedasticity of variance of the model residuals were checked using Shapiro-Wilk and Bartlett test, respectively, and variables that failed the test were analyzed using the non-parametric Kruskal–Wallis test. *Post hoc* comparisons of means were completed with Tukey’s HSD test using the *glht* function of *multcomp* package (Hothorn et al., 2008) in *R*.

Permutational multivariate analysis of variance (PERMANOVA) was used to test the effect of rotation or previous crop on rhizosphere fungal communities using the *adonis* function in the package *vegan*. Permutation test for homogeneity of multivariate dispersions were performed using the function *betadisper* in R. Non-metric multidimensional scaling (NMDS) was used to compare and visualize the effect of rotation or previous crop on the composition of rhizosphere fungal communities using the function *metaMDS* in *vegan.* The *heatmap.2* function in *gplots* (R v.3.0.2) was used to plot the relative abundance of fungal OTUs or fungal genera under different rotation sequences or previous crops. The *mantel* test in the package *vegan* was used to examine the correlation between rhizosphere fungal community composition and soil properties based on the distance matrix. The relative influence of these soil predictors on rhizosphere fungal communities were quantified using the function *varpart*, a RDA-based variance partitioning method. In all tests, the null hypothesis was rejected at *P* ≤ 0.05.

## Results

### Soil Physicochemical Properties and Pea Yield

In 2015, analysis of the soil physicochemical properties revealed that soil Fe, Mn, Cu, PO_4_-P, and pH value differed significantly among rotation sequences (**Supplementary Table S4**). Soil manganese concentration ranged from 11.6 to 39.4 mg kg^-1^, and the highest concentration was obtained in rotations that included one pea rotated with more diverse crops (WLOP and WCOP). The manganese content (31.83 mg kg^-1^) in pea plot of intensified pulse crops rotation (WPLP) was lower than the above rotations but much higher than the other rotations. Concentrations of Fe, PO_4_-P, and Cu also showed similar trends and all were significantly affected by the rotation sequences. Soil pH was low in the rotations WCOP, WLOP, and WPLP. In contrast, impact of crop rotations on soil physicochemical properties was weaker in 2016. Only soil Cu concentration was significantly affected by the rotations (*P* = 0.005) and the highest concentration was obtained in CWCP (**Supplementary Table S4**).

Analysis of previous crop effect showed that more physicochemical properties were affected in 2015 than in 2016. Pea preceded by lentil had a significantly higher concentration of soil NO_3_-N (10.57 mg kg^-1^) whereas the concentration of PO_4_-P was similar to pea preceded by wheat, and concentration of organic carbon was similar to pea preceded by oat (**Supplementary Table S5**). The Ca content showed a completely different trend, and was the lowest in pea preceded by lentil and was the highest in pea preceded by canola. Only Ca content was significantly affected by previous crop in 2016, and pea preceded by wheat had the highest Ca content whereas pea preceded by lentil had the lowest (**Supplementary Table S5**).

**FIGURE 1:**
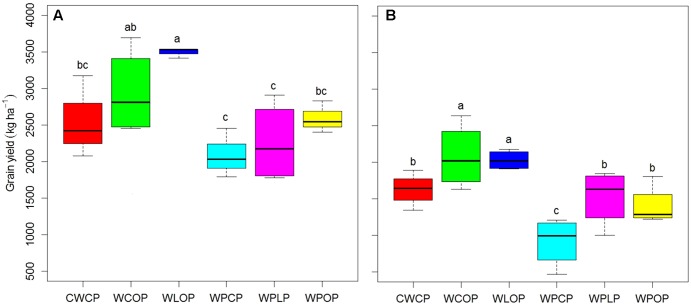
Boxplot showing pea grain yield under various cropping systems in 2015 **(A)** and 2016 **(B)** at Indian Head, SK. Horizontal lines associated with boxes represent the median interquartile range; whiskers represent the range observed. Error bars with different letters indicate a significant difference at *P* < 0.05. Rotation sequence: WCOP, wheat-canola-oat-***pea***; CWCP, canola-wheat-canola-***pea***; WLOP, wheat-lentil-oat-***pea***; WPCP, wheat-pea-canola-***pea***; WPLP, wheat-pea-lentil-***pea****;* WPOP, wheat-pea-oat-***pea***. Italic pea in the rotation indicates pea sequence in each rotation. Same code applies to following figures, where applicable.

**FIGURE 2:**
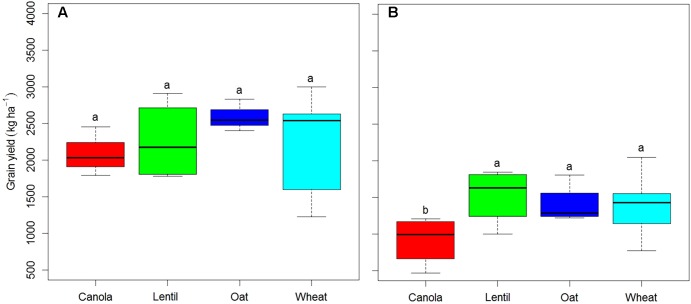
Boxplot showing pea grain yield under various previous crops in 2015 **(A)** and 2016 **(B)** at Indian Head, SK. Horizontal lines associate with boxes represent the median interquartile range; whiskers represent the range observed. Error bars with different letters indicate a significant difference at *P* < 0.05. Previous crop effect was analyzed in the intensified rotation sequence WPCP, WPLP, and WPOP; canola, wpCp, lentil, wpLp, oat, wpOp, and wheat, Wpcp+Wplp+Wpop, the capital letter in these rotation codes indicates the previous crop whose effect on grain yield was tested.

Pea grain yield was significantly affected by rotation sequences in both years (**Figure 1**) and by previous crops in 2016 (**Figure 2**). Rotation sequences with diverse crops (WCOP and WLOP) had comparatively higher grain yield compared to the rotations including two pea or two canola (WPCP, WPLP, WPOP, and CWCP) crops, and this, in both years. Rotations with two pea and canola as break crops (WPCP) had the lowest grain yield. Pea preceded by canola had the lowest grain yield in 2016 while the effect of other previous crops was not significantly different (**Figure 2**). However, average pea grain yield was significantly higher in 2015 than in 2016 (2651 kg and 1596 kg ha^-1^, respectively; *P* < 0.001).

There were significant relationships among soil physicochemical properties, and between pea grain yield and soil properties in the rotation systems, in both years (**Supplementary Tables S6, S7**). Grain yield was negatively correlated with pH (*P* ≤ 0.05) and positively correlated with Fe, Mn, Zn, K, and organic carbon, in 2015. However, the trial showed significant correlation only with Mg concentration (rho = -0.42, *P* ≤ 0.05) in 2016 (**Supplementary Table S7**).

### Fungal Diversity

**FIGURE 3:**
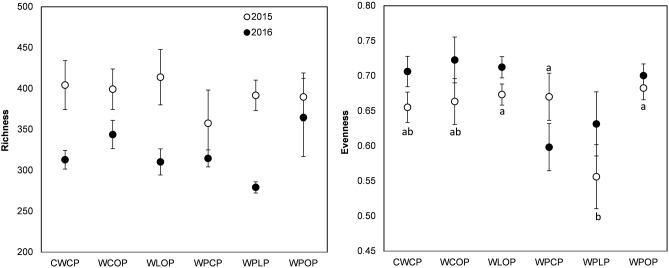
Variation (mean ± SE) of the richness (Chao1 index) and evenness (Pielou’s index) of the fungal community of phase-4 pea rhizosphere as influenced by cropping system in 2015 and 2016. Year influenced richness (*P* < 0.001), but cropping system had no significant effect. Cropping system influenced evenness in 2015. Error bars with different letters indicate a significant difference at *P* < 0.05, according to Tukey HSD.

**FIGURE 4:**
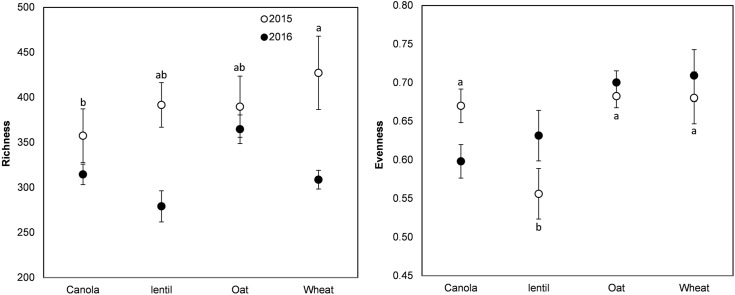
Variation (mean ± SE) of the richness (Chao1 index) and evenness (Pielou’s index) of the fungal community of pea rhizosphere as influenced by previous crops in 2015 and 2016. Year influenced richness (*P* < 0.001), but previous crops had no significant effect. Previous crops influenced evenness in 2015. Error bars with different letters indicate a significant difference at *P* < 0.05, according to Tukey HSD.

The rotation sequence or previous crop had no significant effect on the richness (Chao1 index) of pea rhizosphere fungal communities in 2015 or 2016 but both showed significant effect on fungal evenness (Pielou’s index) in 2015 (**Figures 3, 4**). The rhizosphere fungal community was richer (*P* < 0.001) in 2015 than in 2016. The lowest level of pea rhizosphere fungal evenness was observed in the rotation WPLP or in pea preceded by lentil in 2015.

### Soil Fungal Community Composition

**FIGURE 5:**
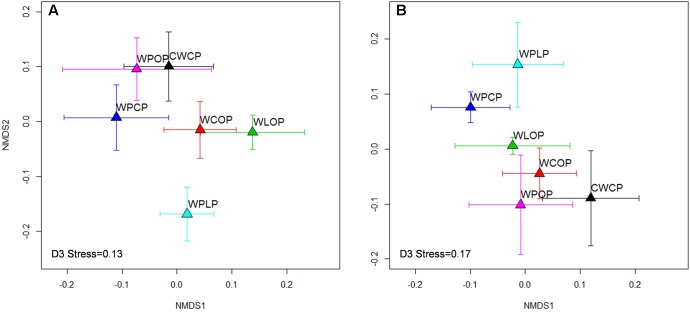
Non-metric multidimensional scaling (NMDS) ordination of rotations on rhizosphere fungal communities in 2015 **(A)** and 2016 **(B)**. Symbols indicate the mean axis coordinates (±SE) for each cropping system. A significant (*P* < 0.001) year effect was observed for fungal community composition and thus the year 2015 and 2016 were presented separately.

The composition of the rhizosphere fungal community was influenced by all factors. Year explained more of the variation than cropping system [year: *P* < 0.001 (*R*^2^ = 0.19) *vs.* cropping system: *P* = 0.03 (*R*^2^ = 0.12)] or previous crop [year: *P* < 0.001 (*R*^2^ = 0.17) *vs.* previous crop: *P* = 0.006 (*R*^2^ = 0.09)]. The effect of cropping system was greater than the effect of previous crop and explained more of the variation in the fungal community both in 2015 and 2016 (**Table 1**). The overall fungal community pattern in different crop rotation sequences was visualized by NMDS ordination based on Bray-Curtis dissimilarity (**Figure 5**). In 2015, a clustering pattern revealed that the WPCP, WPOP, and CWCP rotations had similar fungal communities and clustered together, while they were distinct from the rotation WPLP (**Figure 5A**). In 2016, the pattern was similar to that of 2015, fungal communities in WPLP rotation was distinct from the other rotations (**Figure 5B**).

**Table 1:** PERMANOVA results for the effect of cropping system and previous crop on pea rhizosphere fungal communities in 2015 and 2016.

	2015		2016	
	R^2^	*P*	R^2^	*P*
Rotation	0.28	0.011	0.24	0.048
Previous crop	0.19	0.013	0.17	0.011

**FIGURE 6:**
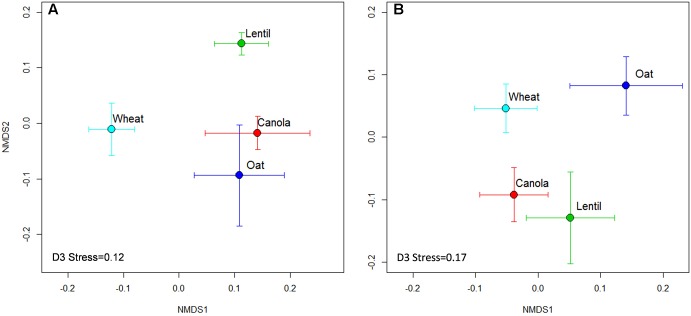
Non-metric multidimensional scaling (NMDS) ordination of previous crops on rhizosphere fungal communities in 2015 **(A)** and 2016 **(B)**. Symbols indicate the mean axis coordinates (±SE) for each crop. A significant (*P* < 0.001) year effect was observed for fungal community composition and thus the year 2015 and 2016 were presented separately.

The composition of the rhizosphere fungal communities was significantly influenced by the previous crop. The NMDS analysis revealed that the rhizosphere fungal communities of pea were distinctive when preceded by lentil, wheat, and oats, both in 2015 and 2016, but varied between years when preceded by canola (**Figure 6**).

**FIGURE 7:**
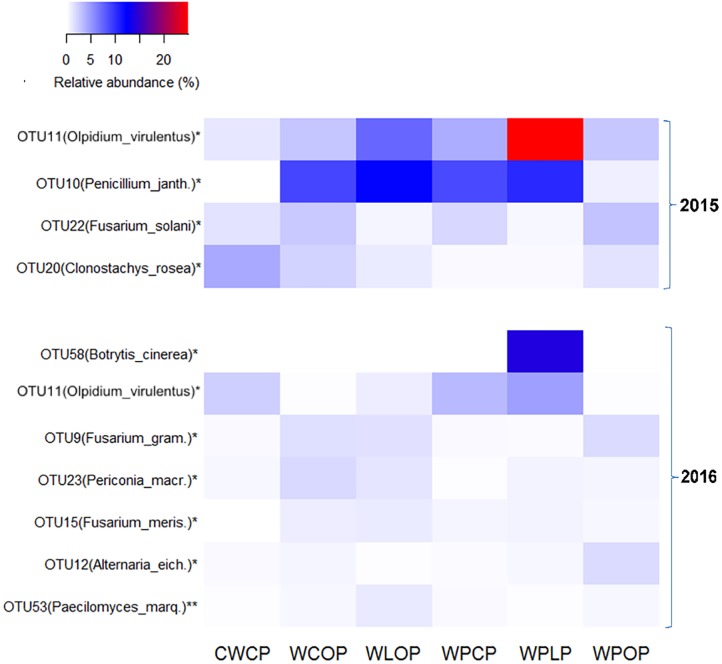
Heatmap showing the most abundant fungal OTUs (>0.2% of total reads) in pea rhizosphere that significantly influenced by cropping system in both 2015 and 2016. OTUs with an ^∗^ indicates a significant difference in relative abundance among the rotation sequences at ^∗^*P* < 0.05 and ^∗∗^*P* < 0.01. *Penicillium_janth., Penicillium janthinellum; Fusarium_gram., Fusarium graminearum; Periconia_macr., Periconia macrospinosa; Fusarium_meris., Fusarium merismoides; Alternaria_eich., Alternaria eichhorniae;* and *Paecilomyces_marq., Paecilomyces marquandii.*

Cropping system had significant effect on the reads of fungal OTUs. OTU11, identified as *Olpidium virulentus*, was represented by the highest proportion of reads in both years. The relative abundance of OTU11 was significantly affected by the rotation sequence (**Figure 7**) and was the highest in WPLP (24.8 and 4.7% of reads in 2015 and 2016, respectively). Rotation WLOP (12.3%) followed by WPLP (10.4%) had the highest relative abundance of OTU10 (*Penicillium janthinellum*) reads and the lowest reads were obtained in the rotation CWCP. Cropping system influence the abundance of *Fusarium* spp., and the relative abundance of both OTU22 (*F. solani*) and OTU9 (*F. graminearum*) were the highest in the rotation WPOP (3.0%). The reads of other OTUs, such as OTU20 (*Clonostachys rosea* f. *catenulata*) were comparatively higher in the rotation CWCP compared to other rotations in 2015, while OTU23 (*Periconia macrospinosa*) and OTU15 (*F. merismoides*) were higher in the rotations with a single pea (WCOP and WLOP) in 2016.

**FIGURE 8:**
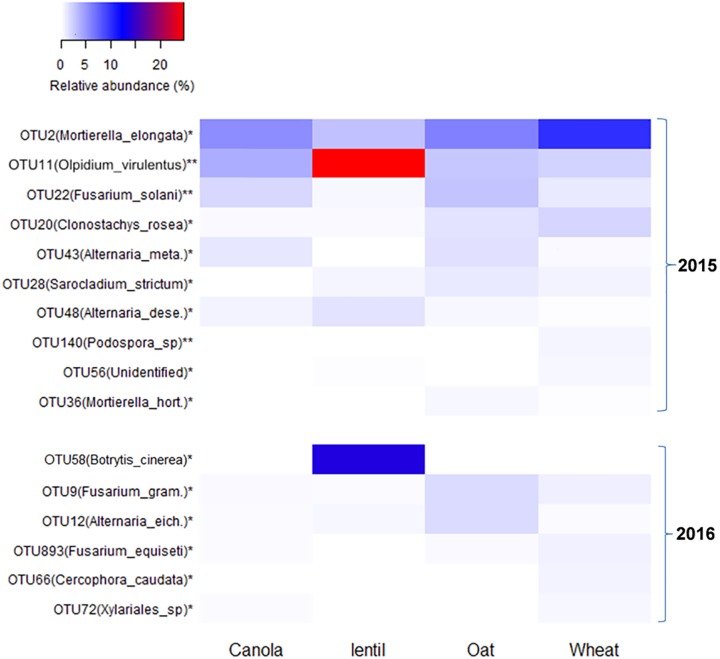
Heatmap showing the most abundant fungal OTUs (>0.2% of total reads) in pea rhizosphere that significantly influenced by previous crop in both 2015 and 2016. OTUs with an ^∗^ indicates a significant difference in relative abundance among previous crops at ^∗^*P* < 0.05 and ^∗∗^*P* < 0.01. *Alternaria_meta., Alternaria_metachromatica; Alternaria dese., Alternaria deserticola; Mortierella hort., Mortierella horticola; Fusarium_gram., Fusarium graminearum;* and *Alternaria_eich., Alternaria_eichhorniae.*

Fungal OTUs were influenced by previous crops in both 2015 and 2016. In 2015, the proportion of the reads of OTU2 [*Mortierella elongata* (NCBI: JF439485.1)] was higher in the rhizosphere of pea preceded by wheat than other previous crops (**Figure 8**). OTU11 (*O. virulentus*) was especially abundant in the rhizosphere of pea following lentil. In 2016, OTU58 [*Botrytis cinerea* (NCBI: MF926241.1)] was particularly high in the rhizosphere of pea following lentil (14.4%) (**Figure 8**). Effects of previous crops on other OTUs, such as OTU9 (*F. graminearum*), OTU12 (*Alternaria eichhorniae*), OTU893 (*F. equiseti*), and OTU66 (*Cerocophora caudata*) were also significant in 2016.

### Fungal Functional Ecological Guilds

**FIGURE 9:**
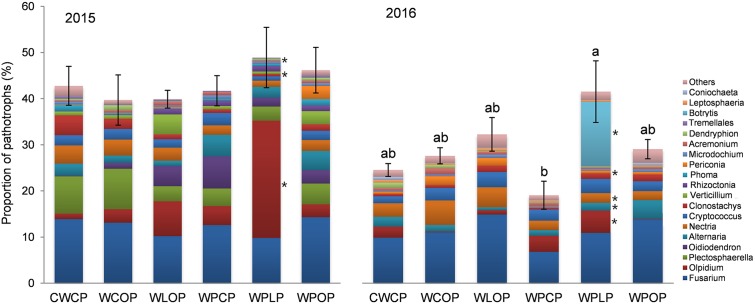
Effect of cropping system on the abundance of fungal genera (>0.25% of total reads) assigned to trophic mode of pathotrophs (mean ± SE) in 2015 and 2016. The effect of cropping system was insignificant in 2015. Bars with different letters indicate a significant difference in the proportion of pathotroph reads between cropping system (*P* < 0.05). Different color bar of fungal taxa with an ^∗^ indicates a significant effect of cropping system on the proportion of pathotrophs’ reads at *P* < 0.05.

The percentage of pathotrophs’ reads in the rhizosphere fungal community differed (*P* < 0.001) among the cropping systems only in 2016 (**Figure 9**). The rotation sequences with two peas and lentil as a break crop (WPLP) had a significantly higher proportion of fungal pathotrophs in the rhizosphere compared to the rotation with two pea crops and canola as break crop (WPCP) (**Figure 9**).

The composition of the pea rhizosphere pathotroph reads was separated into species level to highlight the read abundance of specific pathotrophs. The genus *Fusarium* had the highest relative abundance, both in 2015 (12.3% of total reads) and 2016 (11.2% of total reads) (**Figure 9**). Five *Fusarium* species were identified, namely *F. redolens* (3.25 and 2.7%), *F. solani* (3.09 and 2.5%), *F. avenaceum* (2.62 and 1.5%), *F. graminearum* (1.61 and 2.0%), and *F. oxysporum* (0.8 and 1.0%). *Fusarium solani* reads were more abundant (*P* = 0.006) in WPOP (5.8% of reads) than WLOP (1.35%) and WPLP (1.14%), in 2015. The proportion of *F. graminearum* reads were higher (*P* = 0.016) in WCOP and WPOP than in other rotations in 2016.

*Olpidium* was the second most abundant genus in the pea rhizosphere (7.3% of total reads) in 2015, and its reads were more abundant (*P* = 0.008) in WPLP (25.4%) than in the other cropping systems (**Figure 9**). Cropping system influenced the proportion *Verticillium* and *Dendryphion* in 2015, based on read abundance. For example, *Verticillium* reads were most abundant in WLOP, while *Dendryphion* reads were more abundant (*P* = 0.030) in WCOP than in other cropping systems.

In 2016, *Nectria* (7.3% of total reads) was the most abundant pathotrophic genus after *Fusarium* in the rhizosphere of pea. *Nectria* was more abundant (*P* = 0.010) in WCOP than in rotations with two peas (WPCP, WPLP and WPOP). *Botrytis* reads (2.3% of total reads) was most abundant (*P* = 0.010) in WPLP. Other pathotrophic genera such as *Alternaria* and *Periconia* also varied significantly with cropping system in 2016 (**Figure 9**).

**FIGURE 10:**
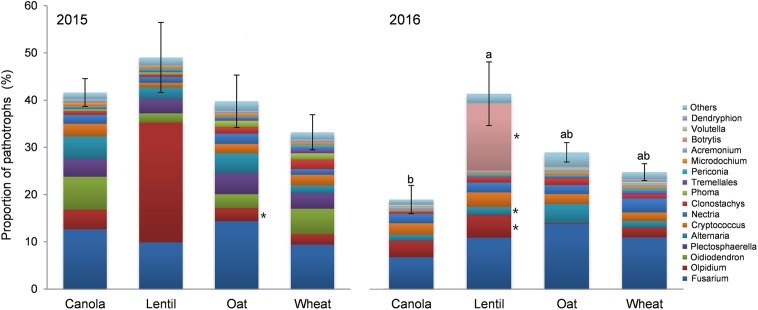
Effect of previous crop on the relative abundance of fungal genera (>0.25% of total reads) assigned to trophic mode of pathotrophs (mean ± SE) in 2015 and 2016. The effect of previous crop was insignificant in 2015. Bars with different letters indicate a significant difference in the relative of pathotroph reads (*P* < 0.05). Different color bar of fungal taxa with an ^∗^ indicates a significant effect of previous crop on the proportion of pathotrophs’ reads at *P* < 0.05.

Effect of previous crop on pea rhizosphere fungal community was also analyzed in respect of functional ecological guilds. Significant variation (*P* = 0.005) in the functional guilds was observed due to the previous crops in 2016 (**Figure 10**). Pea preceded by lentil had the highest proportion of pathotrophs in the rhizosphere and the lowest was in pea preceded by canola. The proportion of the reads of *Fusarium* spp. was the highest in pea rhizosphere influenced by the previous crop both in 2015 and 2016 (10.9 and 10.7% of total reads, respectively) (**Figure 10**). *Fusarium solani* reads (5.78%) were more abundant (*P* = 0.004) in the rhizosphere of pea preceded by oat, while reads were least abundant following lentil (1.14%). *Fusarium graminearum* were more abundant (*P* = 0.021) in pea rhizosphere preceded by oat and wheat than by canola and lentil, in 2016. The relative abundance of the pathotrophic genus *Olpidium* was the second highest OTUs (>0.25% of total reads) in pea rhizosphere in 2015, and the proportion of *Olpidium* reads (24.8%) was significantly higher (*P* = 0.003) in the pea rhizosphere preceded by lentil than preceded by oat and wheat (**Figure 10**). The proportion of *Botrytis* reads was highest in the pea rhizosphere preceded by lentil (14.1%) while the genus *Alternaria* had the highest reads in pea preceded by oat compared to the pea rhizosphere preceded by other crops in 2016.

### Relationship Between Fungal Communities and Soil Properties

The structure of the fungal community in pea rhizosphere was related (*P* = 0.005, *r* = 0.392, *mantel* test) with soil properties in 2015, only. Detailed analysis revealed that pH (*P* < 0.001, *r* = 0.376), soil moisture (*P* = 0.042, *r* = 0.244), Mn (*P* = 0.005, *r* = 0.186), Mg (*P* = 0.041, *r* = 0.291), and Ca (*P* = 0.045, *r* = 0.284) were driving this relationship. This result was also confirmed by redundancy analysis (RDA). Variation partitioning by RDA revealed that soil pH accounted for 9.6%, Mn for 5.4%, Ca for 4.3%, and Mg for 1.90% of the variation observed in the fungal community of pea rhizosphere. However, 78.5% of the variation remained unexplained by soil pH, soil moisture or the abundance of nutrients.

Significant correlations between fungal OTUs and soil properties were also found in 2015 (**Supplementary Table S8**). Spearman correlation analysis revealed that the relative abundance of OTU11 (*O. virulentus*) was positively correlated with the concentrations of soil Mn and PO_4_-P, but the relationship was negative with soil pH. The proportion of OTU10 (*P. janthinellum*) was correlated positively with the concentrations of soil PO_4_-P, Cu, Fe, Mn, and Zn, and negatively with soil pH and Ca concentration. The great correlations between soil properties including pH, Fe, Mn, Zn, PO_4_-P, and Ca, and the relative abundance of other OTUs identified in pea rhizosphere were also observed in 2015 (**Supplementary Table S8**).

## Discussion

We adopted a high throughput sequencing approach to investigate the composition and relative abundance of the pea rhizosphere fungal community in six 4-year cropping systems with different combinations of pea, lentil, hybrid canola, oat, and wheat. Our results showed that climatic factors (e.g., precipitation, temperature) and cropping system had significant effects on the composition of rhizosphere fungal communities, and these effects explained more of the variation than previous crops in the fungal communities. Climatic factors had a strong effect on fungal richness, while cropping systems and previous crops in rotation had strong effects on fungal evenness. Rotations with continuous pulses (i.e., WPLP) or pea preceded by lentil decreased rhizosphere fungal evenness but increased the proportion of fungal pathotrophs, based on partial ITS1 sequences. Among various soil properties we evaluated, soil pH and some physicochemical properties such as moisture, and Mg and Mn concentrations appeared as the best predictors of fungal communities. Rotations with more crop diversity (i.e., WCOP and WLOP) had the highest pea grain yields among the six cropping systems evaluated in the study.

In this study, soil pH varied significantly among the cropping systems, and significant relationship of soil pH with other soil nutrients was observed. With few exceptions, concentration of most of the micronutrients decreased with increasing soil pH (**Supplementary Tables S6, S7**), a trend also reflected in cropping system. The rotations WCOP, WLOP, and WPLP had the lowest pH value and higher concentration of nutrients (e.g., Fe, Mn, Zn, and PO_4_-P) compared to the other rotations. Pea is a leguminous crop and significant proportion of its nitrogen demand is met by biological N_2_-fixation. During N_2_-fixation, pea plants take up more cations (such as K, Na, Ca, and Mg) than anions to balance the organic anions produced in the process of photosynthesis, and release H_3_O^+^ ions into the rhizosphere through roots, leading to acidification of the rhizosphere soil (Haynes, 1983). On the other hand, high organic matter accumulation and decomposition of organic matter can change soil pH in agricultural ecosystem (Haynes, 1983; Xu et al., 2006). In the current study, six rotation systems that included four crop species with different rotation sequences and intensities might influence the composition of particulate soil organic matter and organic matter cycling efficiency, therefore the soil properties. This is probably the reason of pH variation among different rotation sequences in this study.

Soil pH strongly influenced pea rhizosphere fungal community in the current study and similar results were reported in several previous studies (Fujimura and Egger, 2012; Bainard et al., 2017; Degrune et al., 2017). Fungi generally have a limited pH tolerance range and change in pH directly influence fungal community. In this study, for example, the abundance of OTU10 and OTU2 changed significantly with soil pH change (**Supplementary Table S8**). The soil microbiome has a strong relationship with soil physicochemical properties, including nutrient availability, ion exchange capacity, and organic carbon and micronutrient contents (Berg and Smalla, 2009; Yang et al., 2017). In agreement with previous findings, the pea rhizosphere microbiome in this study was significantly influenced by different micronutrients. Significant correlation between rhizosphere fungal community composition and relative abundance with soil moisture level was also observed (**Supplementary Table S8**). Peas are shallow-rooted crops with approximately 77–85% of their roots located in the top 0 to 40 cm soil layer (Liu et al., 2010). This morphological feature may change the living environment (such as water content, pH, nutrients, soil aggregate stability etc.) relative to deep-rooted crops with fibrous root systems such as wheat, and impact the activity and composition of the fungal communities (Hinsinger et al., 2006; Bainard et al., 2017).

Generally, rhizosphere microbial communities are selectively affected by plant root exudates, which largely determine the composition and activity of the microbial community, including fungi (Jones et al., 2009; Dennis et al., 2010). Rhizosphere microbiomes also influence plant growth and development by secreting a wide array of substances. Some microbiota or fungi provide beneficial services to their host, including breaking down crop residues, facilitating nutrient uptake from surrounding soil and indirect disease protection by negatively affecting plant pathogens (Saldajeno et al., 2008; Berendsen et al., 2012; Fujimura and Egger, 2012; Hermosa et al., 2012).

Based on the analysis of partial ITS1 sequences, we found the proportion of several fungal pathotrophs in the pea rhizosphere was significantly influenced by cropping system or preceding crops. Pathotrophs form one of three broadly defined trophic modes (Tedersoo et al., 2014). Pathotrophs have the ability to acquire nutrients by harming host cells (Nguyen et al., 2016). *Fusarium* spp. is abundant members of this guild. They are ubiquitous and generally abundant soil fungi involved in important crop diseases (Fernandez, 2007; Fernandez et al., 2014). *Fusarium* spp. isolated from pulses in Saskatchewan includes *F. avenaceum, F. solani, F. redolens, F. oxysporum, F. graminearum (Gibberella zeae), F. equiseti, F. culmorum*, and *F. poae.* These fusaria are non-specialized pathogens causing crop root rot and head blight (Alberta Pulse Growers et al., 2016). It was reported that *F. redolens* and *F. avenaceum* are the most virulent *Fusarium* species on pea, and they are common in the Prairie Ecozone of Saskatchewan (Fernandez, 2007; Esmaeili Taheri et al., 2011; Bainard et al., 2017). The abundance of *F. solani*, the main root rot pathogen of pea, was increased with pea intensity (Oyarzun et al., 1993; Bainard et al., 2017). In both years of our study, *Fusarium* was the most abundant fungal genera in pea rhizosphere. It was mainly composed of five species identified as *F. redolens, F. solani, F. avenaceum, F. graminearum*, and *F. oxysporum.* The finding suggests that *Fusarium* is a common root rot pathogen in the study area, which is in agreement with previous studies (Fernandez, 2007; Gossen et al., 2016). We observed that rotations including lentil critically reduced the relative abundance of *F. solani* reads in 2015, while the abundance of *F. graminearum* in pea preceded by oats was fivefold higher than in other rotations in 2016 (**Figure 7**). This indicated the importance of crop species selection on the prevalence of pathotrophs in cropping systems. We did not investigate the pathogenicity of the *Fusarium* species in this study. However, it was previously shown that *Fusarium* root rot severity increased with continuous-pea (Nayyar et al., 2009).

*Olpidium* spp. is zoosporic fungi belonging to the division Chytridiomycota and obligates intracellular parasites of roots (Rochon, 2009; Yu et al., 2012). *O. virulentus* is one of several major vectors of plant virus transmission (Campbell and Fry, 1966; Campbell and Sim, 1994; Maccarone et al., 2010; Maccarone, 2013) and is currently the only known fungal species that transmit plant viruses (Singh et al., 2008; Rochon, 2009). OTU11 sequence was identified as *Olpidium brassicae* using the UNITE fungal ITS database, and we manually BLAST it in NCBI and identified as *O. virulentus* (GenBank: KY905661.1). Lay et al. (2018) recently clarify that *O. brassicae* infects only the *Brassicaceae*, which includes canola, whereas *O. virulentus* infects a wide variety of hosts. Maccarone et al. (2010) compared *O. virulentus* and *O. brassicae* isolates from Brassicaceae plants, lettuce, melons, cucumbers, and other species through the amplification of the ITS regions and showed that members of the *O. virulentus* subgroup can be found in different crops and are not restricted by geographical barriers. We found a high abundance of *O. virulentus* in the rotation of intensified pulses rotated with wheat (WPLP) in both years (**Figures 9, 10**). It is possible that rotation with pea and lentil creates a more favorable environment for *O. virulentus* reproduction than the other rotations in this study. However, we did not observe any disease symptom linked to *Olpidium* spp. or the associated virus, probably because of the complexity of the interactions of *Olpidium* vector with viruses and plant hosts (Maccarone, 2013). The high abundance of *O. virulentus* in both years suggests that this soil borne fungal species or associated viruses are prevalent in local farmlands. To our knowledge, this is the first report of *O. virulentus* in pea rhizosphere in western Canadian prairies.

*Botrytis* is another group of pathogens able to cause seedling diseases in pulses (Alberta Pulse Growers et al., 2016). The relative abundance of OTU58, identified as *B. cinerea* (NCBI: MF926241.1), was highest in 2016. *B. cinerea* is an ubiquitous fungal pathogen causing gray rot on a large number of economically important agricultural and horticultural crops, including pea (Jarvis, 1977; Keller et al., 2003; Kulakiotu et al., 2004). The high abundance of *B. cinerea* in the rotation WPLP underscores the risk of new disease development in intensified pulse based rotation systems.

The rotational benefits of field pea include enhanced soil water conservation, improved soil N availability, and increased system productivity (Gan et al., 2015). The possibility of stimulation of beneficial microorganisms by pea was also proposed previously (Lupwayi and Kennedy, 2007; Yang et al., 2013). We found only a few weak relationships between fungal OTU and plant productivity, despite the presence of potentially beneficial soil fungi. *Penicillium* spp. is one of the most common fungi present in a diverse range of habitats. *Penicillium* spp. plays important roles as decomposers of organic materials (Visagie et al., 2014). Many *Penicillium* spp. showed wide range of tolerance to temperature, pH, and salinity (Pandey et al., 2008). Some *Penicillium* spp. can release fixed phosphorus in the soil and make it available to plants (Morales et al., 2007; Pandey et al., 2008; Saber et al., 2009; Sahoo and Gupta, 2014) or serve both as disease control agent and biofertilizer (Ownley and Benson, 1992; Dong et al., 2006). For example, dry mycelium of *P. chrysogenum* was successfully used for the control of Fusarium wilt and Verticillium wilt as well as nutrient source in cotton production (Dong et al., 2006). *P. janthinellum* was reported to improve P availability to coffee plants (Posada et al., 2013). The proportion of reads of *P. janthinellum* (NCBI: HQ607930.1) was the highest among the *Penicillium* species in our study, and it was especially abundant in the pulse dominant rotations WPLP and WLOP. The positive relationship between the abundance of *P. janthinellum* and P availability that we observed, suggests that *P. janthinellum* may benefit pea growth by enhancing inorganic phosphate availability in the root zone. Further study in this area may unfold the beneficial effect of *P. janthinellum* on pea or crop production system as a whole.

The fungus *C. rosea* f. *catenulate* is a mycoparasite antagonizing a range of fungal plant pathogens, including *Sclerotinia sclerotiorum, Rhizoctonia solani*, and *Fusarium* spp. (Appuhn and Joergensen, 2006; Chatterton and Punja, 2009). The abundance of this fungus in CWCP suggests that the inclusion of canola in cropping systems might improve soil biological quality. Members of the genus *Mortierella* within the family *Mortierellaceae* are filamentous fungi commonly found in soil. One of the species, *M. elongata* (OTU2), was specifically abundant in the rhizosphere of intensive pea rotation preceded by wheat in our experiment (**Figure 8**). Previous study suggested that the species of *Mortierella* may benefit wheat yield (Borrell et al., 2017). The abundance pattern of beneficial or pathogenic fungi revealed that each fungus has its own preferred growth environment, which varies with cropping system.

Diversifying cropping systems with pulses become an important farming practice that enhances system productivity, in the Canadian prairies (Gan et al., 2015). This study demonstrates the influence of the plants included in pea-intensified cropping systems on the properties of the soil and on the proportion fungal pathotrophs in pea rhizosphere. Cropping systems change soil physicochemical properties, which in turn influences rhizosphere fungal communities. This relationship was confirmed in 2015 by *mantel* test, which revealed that soil fungal community was significantly correlated with soil properties. However, the effect of rotation sequence on soil physiochemical properties was insignificant in 2016, and the influence of rotation on the composition of the pea rhizosphere community was weaker than in 2015. This phenomenon suggests that other factors, such as the soil environmental conditions (e.g., amount of precipitation and its seasonal distribution, temperature), had a greater effect on the composition and abundance of pea rhizosphere fungal community.

This study revealed the influence of 4-year cropping systems on the rhizosphere fungal community. The selection of crops and their sequence in rotation explained more of the variation in the fungal communities of pea rhizosphere in rotation phase-4 than the previous crop, at least partly due to modified soil physicochemical properties. Among soil properties, pH was the main driver of fungal community shift. Increasing the frequency of pea or pulses in the cropping system decreased fungal evenness and led to an apparent build-up of pathogens in the soil compared to diversified rotations. Frequent canola also increased the abundance of fungal pathotrophs. The build-up of fungal pathogens in the rhizosphere or in soil is a risk of disease development and yield losses, and farmers should avoid growing pulses or canola in consecutive years. Pea grown in diversified cropping systems including wheat, canola, lentil, and oat had consistently higher grain yields than cropping system including two repeated crops of canola or pea. Results from this study confirm the value of the current practice of crop diversification for improved soil properties and increased system productivity. This study also highlighted the importance of crop selection in cropping systems to maintain a healthy soil biology and ensure the productivity and profitability of crop production systems.

## Author Contributions

YG and WM conceived the idea, designed the project, and secured funds for the implementation of the project. WM performed the field work. YN conducted the laboratory tests and analyses guided by LB, CH, and YG. YN, ZH, and CH wrote the paper. All authors contributed to data interpretation and manuscript writing.

## Conflict of Interest Statement

The authors declare that the research was conducted in the absence of any commercial or financial relationships that could be construed as a potential conflict of interest.
