# Supplementary material for: Intensified Pulse Rotations Buildup Pea Rhizosphere Pathogens in Cereal and Pulse Based Cropping Systems
Source: Front Microbiol. 2018 Aug 23;9:1909. doi: 10.3389/fmicb.2018.01909 (PMC6115495; doi:10.3389/fmicb.2018.01909)
Supplement: Supplementary file 1 [file Table_1.DOCX]

Supplementary Material

Intensified pulse rotations buildup pea rhizosphere pathogens in cereal and pulse based cropping systems

Yining Niu, Luke D. Bainard, Zakir Hossain, William E. May, Chantal Hamel, Yantai Gan*

*** Correspondence:** [yantai.gan@agr.gc.ca](mailto:yantai.gan@agr.gc.ca)

Table S1. Crop frequencies and sequences used in 4-yr cropping system

| Rotation code | Crop sequence | | | |
| --- | --- | --- | --- | --- |
|  | Yr-1 | Yr-2 | Yr-3 | Yr-4 |
| CWCP | Canola | Wheat | Canola | Pea |
| WCOP | Wheat | Canola | Oat | Pea |
| WLOP | Wheat | Lentil | Oat | Pea |
| WPCP | Wheat | Pea | Canola | Pea |
| WPLP | Wheat | Pea | Lentil | Pea |
| WPOP | Wheat | Pea | Oat | Pea |
