# Supplementary material for: Intensified Pulse Rotations Buildup Pea Rhizosphere Pathogens in Cereal and Pulse Based Cropping Systems
Source: Front Microbiol. 2018 Aug 23;9:1909. doi: 10.3389/fmicb.2018.01909 (PMC6115495; doi:10.3389/fmicb.2018.01909)
Supplement: Supplementary file 2 [file Table_2.DOCX]

Supplementary Material

Intensified pulse rotations buildup pea rhizosphere pathogens in cereal and pulse based cropping systems

Yining Niu, Luke D. Bainard, Zakir Hossain, William E. May, Chantal Hamel, Yantai Gan*

*** Correspondence:** [yantai.gan@agr.gc.ca](mailto:yantai.gan@agr.gc.ca)

Table S2. Crop variety, target density, field mortality and target N fertilizer rate in 4-yr rotation systems

| Crop | Cultivar | Target density  (seeds m^−2^) | Field mortality  (seeds m^−2^) | Target N rate^*^  (kg ha^-1^) |  | P, K, S |
| --- | --- | --- | --- | --- | --- | --- |
| Wheat | Unity | 250 | 10 | 110 |  | Applied based on soil test recommendations from fall soil samples |
| Canola | L252 LL | 75 | 40 | 120 |  |  |
| Oat | Triactor | 375 | 20 | 80 |  |  |
| Field pea | Golden | 80 | 25 | - |  |  |
| Lentils | Maximum | 130 | 25 | - |  |  |

^*^Target N rate = total soil N (Kjeldahl N digestion method) at top 0-60 cm soil layer + fertilizer N.
