# Supplementary material for: Intensified Pulse Rotations Buildup Pea Rhizosphere Pathogens in Cereal and Pulse Based Cropping Systems
Source: Front Microbiol. 2018 Aug 23;9:1909. doi: 10.3389/fmicb.2018.01909 (PMC6115495; doi:10.3389/fmicb.2018.01909)
Supplement: Supplementary file 3 [file Table_3.DOCX]

Supplementary Material

Intensified pulse rotations buildup pea rhizosphere pathogens in cereal and pulse based cropping systems

Yining Niu, Luke D. Bainard, Zakir Hossain, William E. May, Chantal Hamel, Yantai Gan*

*** Correspondence:** [yantai.gan@agr.gc.ca](mailto:yantai.gan@agr.gc.ca)

Table S3. Average monthly temperature and precipitation during the crop growing season at the experiment site in Indian Head, Saskatchewan

| Month | Mean temperature (°C) | | |  | Precipitation (mm) | | |
| --- | --- | --- | --- | --- | --- | --- | --- |
|  | 2015 | 2016 | LTM |  | 2015 | 2016 | LTM |
| May | 10.0 | 12.8 | 10.8 |  | 15.6 | 74.7 | 49.0 |
| June | 16.2 | 16.9 | 15.8 |  | 38.3 | 50.2 | 77.4 |
| July | 18.1 | 17.6 | 18.2 |  | 94.6 | 107.9 | 63.8 |
| August | 17.0 | 16.9 | 17.4 |  | 58.8 | 21.9 | 51.2 |
| May-Aug. | 15.3 | 16.1 | 15.6 |  | 207.3 | 254.7 | 241.4 |
| ^*^LTM, the long-term means (1981 to 2010, Environment Canada). | | | | | | | |
