# Supplementary material for: Intensified Pulse Rotations Buildup Pea Rhizosphere Pathogens in Cereal and Pulse Based Cropping Systems
Source: Front Microbiol. 2018 Aug 23;9:1909. doi: 10.3389/fmicb.2018.01909 (PMC6115495; doi:10.3389/fmicb.2018.01909)
Supplement: Supplementary file 4 [file Table_4.DOCX]

Supplementary Material

Intensified pulse rotations buildup pea rhizosphere pathogens in cereal and pulse based cropping systems

Yining Niu, Luke D. Bainard, Zakir Hossain, William E. May, Chantal Hamel, Yantai Gan*

*** Correspondence:** [yantai.gan@agr.gc.ca](mailto:yantai.gan@agr.gc.ca)

Table S4. Effect of rotation sequence on soil physicochemical properties at pea flowering stage in 2015 and 2016, Indian Head, SK. Bold face indicates statistical significance (*P* < 0.05, *N* = 4)

| Soil properties  / Grain yield | Rotation | | | | | | P-value |
| --- | --- | --- | --- | --- | --- | --- | --- |
|  | CWCP | WCOP | WLOP | WPCP | WPLP | WPOP |  |
| 2015 |  |  |  |  |  |  |  |
| pH | **7.19±0.21** | **6.33±0.14** | **6.37±0.21** | **6.83±0.36** | **6.36±0.16** | **6.92±0.21** | **0.014** |
| EC (mS) | 1.86±0.65 | 1.65±0.60 | 1.27±0.18 | 3.09±1.24 | 1.74±0.66 | 2.32±0.75 | ns |
| Fe (mg kg^-1^) | **4.42±1.71** | **38.08±15.08** | **42.94±19.9** | **11.15±7.22** | **24.49±7.78** | **8.92±3.58** | **0.020** |
| Mn (mg kg^-1^) | **11.61±2.71** | **39.39±8.33** | **36.92±11.55** | **19.12±10.32** | **31.83±6.22** | **15.73±5.92** | **0.021** |
| Cu (mg kg^-1^) | **1.05±0.12** | **1.45±0.19** | **1.48±0.26** | **1.02±0.09** | **1.32±0.09** | **1.19±0.04** | **0.033** |
| Zn (mg kg^-1^) | 1.58±0.32 | 2.36±0.39 | 2.15±0.36 | 1.89±0.39 | 2.29±0.39 | 1.58±0.24 | ns |
| PO_4_-P (mg kg^-1^) | **13.65±2.44** | **28.28±6.76** | **24.1±4.62** | **18.05±4.61** | **26.40±4.02** | **10.21±2.42** | **0.022** |
| K (g kg^-1^) | 0.29±0.06 | 0.29±0.03 | 0.29±0.01 | 0.23±0.02 | 0.31±0.03 | 0.3±0.32 | ns |
| Mg (g kg^-1^) | 0.80±0.16 | 0.59±0.04 | 0.88±0.27 | 1.22±0.49 | 0.67±0.06 | 1.13±0.49 | ns |
| Ca (g kg^-1^) | 3.30±0.66 | 2.34±0.03 | 2.75±0.34 | 3.64±0.67 | 2.58±0.23 | 3.11±0.60 | ns |
| NO_3_-N (mg kg^-1^) | 6.70±0.85 | 6.35±1.86 | 7.12±3.15 | 6.64±1.03 | 10.57±1.75 | 6.20±0.46 | ns |
| TN^b^ (%^1^) | 1.75±0.02 | 1.93±0.01 | 2.03±0.02 | 2.00±0.02 | 2.18±0.01 | 2.05±0.02 | ns |
| OC^c^ (%) | 21.50±0.11 | 23.38±0.27 | 26.15±0.35 | 23.93±0.2 | 26.08±0.18 | 25.63±0.03 | ns |
| TC^d^ (%) | 24.73±0.29 | 26.55±0.20 | 28.60±0.30 | 27.33±0.17 | 30.68±0.15 | 27.00±0.12 | ns |
| Moisture (%) | 17.24±1.04 | 17.19±1.48 | 20.86±0.69 | 19.47±2.19 | 18.23±0.85 | 20.98±2.28 | ns |
| 2016 |  |  |  |  |  |  |  |
| pH | 7.01±0.15 | 7.07±0.27 | 7.12±0.12 | 7.18±0.19 | 7.05±0.2 | 7.19±0.21 | ns |
| EC (mS) | 1.66±0.95 | 0.69±0.09 | 0.82±0.18 | 1.21±0.50 | 1.11±0.44 | 0.88±0.16 | ns |
| Fe (mg kg^-1^) | 27.77±10.23 | 29.74±16.98 | 21.38±7.3 | 27.86±13.28 | 26.76±12.01 | 23.72±7.93 | ns |
| Mn (mg kg^-1^) | 33.81±7.73 | 30.34±10.29 | 27.6±7.71 | 28.11±10.48 | 28.26±7.25 | 29.07±7.81 | ns |
| Cu (mg kg^-1^) | **2.65±0.88** | **1.28±0.16** | **1.41±0.16** | **1.33±0.15** | **1.63±0.16** | **1.54±0.26** | **0.005** |
| Zn (mg kg^-1^) | 1.03±0.22 | 1.14±0.4 | 0.79±0.22 | 0.93±0.31 | 0.85±0.17 | 1.02±0.30 | ns |
| PO_4_-P (mg kg^-1^) | 5.01±1.67 | 3.89±0.42 | 3.94±0.27 | 4.68±0.86 | 4.31±1.06 | 5.19±0.86 | ns |
| K (g kg^-1^) | 0.27±0.03 | 0.28±0.06 | 0.33±0.08 | 0.30±0.07 | 0.31±0.05 | 0.33±0.04 | ns |
| Mg (g kg^-1^) | 0.70±0.09 | 0.55±0.04 | 0.61±0.02 | 0.64±0.06 | 0.77±0.23 | 0.80±0.17 | ns |
| Ca (g kg^-1^) | 2.61±0.29 | 2.77±0.35 | 2.82±0.50 | 3.41±0.54 | 2.40±0.15 | 3.20±0.41 | ns |
| NO_3_-N (mg kg^-1^) | 3.42±0.51 | 3.81±0.42 | 3.86±0.27 | 4.60±0.86 | 4.56±0.90 | 5.11±0.86 | ns |
| TN (g kg^-1^) | 1.78±0.23 | 1.88±0.21 | 1.63±0.15 | 1.78±0.23 | 1.70±0.17 | 1.93±0.11 | ns |
| OC (g kg^-1^) | 21.13±2.61 | 23.13±2.16 | 19.40±2.02 | 20.33±1.23 | 20.75±1.99 | 22.2±1.43 | ns |
| TC (g kg^-1^) | 22.7±3.15 | 25.43±2.77 | 20.78±1.70 | 25.88±3.46 | 22.38±2.08 | 26.35±2.16 | ns |
| Moisture (%) | 19.57±1.75 | 19.95±2.25 | 18.81±1.53 | 20.19±1.43 | 18.8±2.49 | 20.57±1.15 | ns |

^*^ns, not significant at *P* < 0.05;

^*^EC, Electronic Conductivity; TN, Total Nitrogen; OC, Organic Carbon; TC, Total Carbon;

^*^Rotation sequence: WCOP = wheat-canola-oat-***pea***; CWCP= canola-wheat-canola-***pea***; WLOP=wheat-lentil-oat-***pea***; WPCP= wheat-pea-canola-***pea***; WPLP=wheat-pea-lentil-***pea;*** WPOP=wheat-pea-oat-***pea***. Italic pea in the rotation indicates pea sequence in each rotation.
