# Supplementary material for: Intensified Pulse Rotations Buildup Pea Rhizosphere Pathogens in Cereal and Pulse Based Cropping Systems
Source: Front Microbiol. 2018 Aug 23;9:1909. doi: 10.3389/fmicb.2018.01909 (PMC6115495; doi:10.3389/fmicb.2018.01909)
Supplement: Supplementary file 5 [file Table_5.DOCX]

Supplementary Material

Intensified pulse rotations buildup pea rhizosphere pathogens in cereal and pulse based cropping systems

Yining Niu, Luke D. Bainard, Zakir Hossain, William E. May, Chantal Hamel, Yantai Gan*

*** Correspondence:** [yantai.gan@agr.gc.ca](mailto:yantai.gan@agr.gc.ca)

Table S5. Effect of previous crop on soil physicochemical properties at pea flowering stage in 2015 and 2016, in Indian Head, SK. Bold face indicates statistical significance (*P* < 0.05, *N* = 4)

| P-value | Previous crop | | | | P-value |
| --- | --- | --- | --- | --- | --- |
|  | Canola | Lentil | Oat | Wheat |  |
| 2015 |  |  |  |  |  |
| pH | 6.83±0.36 | 6.36±0.16 | 6.92±0.21 | 6.82±0.20 | ns |
| EC (mS) | 3.09±1.24 | 1.74±0.66 | 2.32±0.75 | 1.79±0.60 | ns |
| Fe (mg kg^-1^) | 11.15±7.22 | 24.49±7.78 | 8.92±3.58 | 19.73±10.88 | ns |
| Mn (mg kg^-1^) | 19.12±10.32 | 31.83±6.22 | 15.73±5.92 | 22.06±8.19 | ns |
| Cu (mg kg^-1^) | 1.02±0.09 | 1.32±0.09 | 1.19±0.04 | 1.16±0.12 | ns |
| Zn (mg kg^-1^) | 1.89±0.39 | 2.29±0.39 | 1.58±0.24 | 1.45±0.22 | ns |
| PO_4_-P (mg kg^-1^) | **18.05±4.61** | **26.4±4.02** | **10.21±2.42** | **27.08±4.55** | **0.001** |
| K (g kg^-1^) | 0.23±0.02 | 0.31±0.03 | 0.30±0.03 | 0.19±0.01 | ns |
| Mg (g kg^-1^) | 1.22±0.49 | 0.67±0.06 | 1.13±0.49 | 0.77±0.17 | ns |
| Ca (g kg^-1^) | **3.64±0.67** | **2.58±0.23** | **3.101±0.6** | **3.13±0.26** | **0.007** |
| NO_3_-N (mg kg^-1^) | **6.64±1.03** | **10.57±1.75** | **6.20±0.46** | **5.61±0.62** | **0.015** |
| TN^b^ (%^1^) | 2.00±0.16 | 2.18±0.06 | 2.05±0.16 | 1.82±0.06 | ns |
| OC^c^ (%) | **23.93±2.01** | **26.08±1.84** | **25.63±0.32** | **22.66±0.60** | **0.012** |
| TC^d^ (%) | 27.33±1.68 | 30.68±1.48 | 27.00±1.23 | 25.43±0.59 | ns |
| Moisture (%) | 19.47±2.19 | 18.23±0.85 | 20.98±2.28 | 16.40±0.81 | ns |
| 2016 |  |  |  |  |  |
| pH | 7.18±0.37 | 7.05±0.40 | 7.19±0.41 | 7.32±0.22 | ns |
| EC (mS) | 1.21±1.00 | 1.11±0.88 | 0.88±0.32 | 1.45±0.95 | ns |
| Fe (mg kg^-1^) | 27.86±26.55 | 26.76±24.01 | 23.72±15.86 | 15.23±6.15 | ns |
| Mn (mg kg^-1^) | 28.11±20.96 | 28.26±14.51 | 29.07±15.62 | 20.97±4.82 | ns |
| Cu (mg kg^-1^) | 1.33±0.31 | 1.63±0.32 | 1.54±0.52 | 1.66±0.89 | ns |
| Zn (mg kg^-1^) | 0.93±0.63 | 0.85±0.34 | 1.02±0.59 | 0.82±0.33 | ns |
| PO_4_-P (mg kg^-1^) | 4.68±1.73 | 4.31±2.12 | 5.19±1.71 | 5.10±2.73 | ns |
| K (g kg^-1^) | 0.30±0.13 | 0.31±0.10 | 0.33±0.08 | 0.30±0.07 | ns |
| Mg (g kg^-1^) | 0.64±0.12 | 0.77±0.46 | 0.80±0.34 | 0.60±0.16 | ns |
| Ca (g kg^-1^) | **3.41±1.08** | **2.40±0.29** | **3.20±0.81** | **3.66±0.90** | **0.018** |
| NO_3_-N (mg kg^-1^) | 4.60±1.73 | 4.56±1.79 | 5.11±1.71 | 5.02±2.73 | ns |
| TN^b^ (%^1^) | 1.78±0.46 | 1.70±0.35 | 1.93±0.22 | 1.80±0.30 | ns |
| OC^c^ (%) | 20.33±2.45 | 20.75±3.99 | 22.20±2.87 | 21.19±4.10 | ns |
| TC^d^ (%) | 25.88±6.92 | 22.38±4.16 | 26.35±4.31 | 24.17±5.39 | ns |
| Moisture (%) | 20.19±2.85 | 18.80±4.97 | 20.57±2.30 | 20.00±2.16 | ns |

^*^ns, not significant at *P* < 0.05;

^*^EC, Electronic Conductivity; TN, Total Nitrogen; OC, Organic Carbon; TC, Total Carbon;

^*^Previous crop effect was analyzed in the intensified rotation sequence of WPCP, WPLP and WPOP, canola=wpCp, lentil= wpLp, oat=wpOp and wheat=Wpcp+Wplp+Wpop, the capital letter in these rotation codes indicates the previous crop whose effects on soil properties were tested.
