# Supplementary material for: Intensified Pulse Rotations Buildup Pea Rhizosphere Pathogens in Cereal and Pulse Based Cropping Systems
Source: Front Microbiol. 2018 Aug 23;9:1909. doi: 10.3389/fmicb.2018.01909 (PMC6115495; doi:10.3389/fmicb.2018.01909)
Supplement: Supplementary file 6 [file Table_6.DOCX]

Supplementary Material

Intensified pulse rotations buildup pea rhizosphere pathogens in cereal and pulse based cropping systems

Yining Niu, Luke D. Bainard, Zakir Hossain, William E. May, Chantal Hamel, Yantai Gan*

*** Correspondence:** [yantai.gan@agr.gc.ca](mailto:yantai.gan@agr.gc.ca)

Table S6. Spearman correlations between the assessed soil physicochemical properties or pea grain yield with soil physicochemical properties in 2015.

|  | pH | EC | Fe | Mn | Cu | Zn | PO_4_-P | K | Mg | Ca | NO_3_-N | TN | OC | TC | Moisture |
| --- | --- | --- | --- | --- | --- | --- | --- | --- | --- | --- | --- | --- | --- | --- | --- |
| EC | 0.07 |  |  |  |  |  |  |  |  |  |  |  |  |  |  |
| Fe | -0.90*** | -0.16 |  |  |  |  |  |  |  |  |  |  |  |  |  |
| Mn | -0.94*** | -0.2 | 0.97*** |  |  |  |  |  |  |  |  |  |  |  |  |
| Cu | -0.57** | -0.05 | 0.75*** | 0.68*** |  |  |  |  |  |  |  |  |  |  |  |
| Zn | -0.72*** | -0.35 | 0.81*** | 0.81*** | 0.42* |  |  |  |  |  |  |  |  |  |  |
| PO_4_-P | -0.48* | -0.29 | 0.41* | 0.50* | 0.25 | 0.46* |  |  |  |  |  |  |  |  |  |
| K | -0.3 | -0.3 | 0.24 | 0.25 | 0.03 | 0.37 | -0.01 |  |  |  |  |  |  |  |  |
| Mg | 0.37 | 0.63*** | -0.54** | -0.50* | -0.36 | -0.61** | -0.23 | -0.22 |  |  |  |  |  |  |  |
| Ca | 0.69*** | 0.35 | -0.83*** | -0.83*** | -0.54** | -0.73*** | -0.27 | -0.41* | 0.67*** |  |  |  |  |  |  |
| NO_3_-N | 0.12 | 0.01 | 0.06 | 0.07 | 0.24 | 0.21 | 0.07 | -0.11 | 0.07 | -0.05 |  |  |  |  |  |
| TN | -0.19 | 0.06 | 0.19 | 0.2 | 0.05 | 0.42* | 0.24 | 0.24 | 0.13 | -0.17 | 0.33 |  |  |  |  |
| OC | -0.47* | 0.02 | 0.52** | 0.51* | 0.41* | 0.62** | 0.28 | 0.4 | -0.17 | -0.39 | 0.24 | 0.61** |  |  |  |
| TC | -0.23 | -0.09 | 0.32 | 0.31 | 0.17 | 0.56** | 0.22 | 0.33 | -0.03 | -0.25 | 0.42* | 0.86*** | 0.69*** |  |  |
| Moisture | 0.05 | 0.23 | -0.01 | -0.07 | 0.15 | 0.02 | -0.21 | 0.04 | 0.44* | 0.19 | 0.28 | 0.41* | 0.34 | 0.49* |  |
| Grain yield | -0.54* | -0.39 | 0.52* | 0.53* | 0.35 | 0.61** | 0.23 | 0.55* | -0.35 | -0.38 | -0.05 | 0.26 | 0.59** | 0.35 | 0.39 |

^*^ EC, Electronic Conductivity; TN, Total Nitrogen; OC, Organic Carbon; TC, Total Carbon;

^*^Values followed with an * indicates a significant correlation between the assessed soil physicochemical properties at **P* < 0.05, ***P* < 0.01 and ****P* < 0.001, *N* = 24.
