# Supplementary material for: Intensified Pulse Rotations Buildup Pea Rhizosphere Pathogens in Cereal and Pulse Based Cropping Systems
Source: Front Microbiol. 2018 Aug 23;9:1909. doi: 10.3389/fmicb.2018.01909 (PMC6115495; doi:10.3389/fmicb.2018.01909)
Supplement: Supplementary file 7 [file Table_7.DOCX]

Supplementary Material

Intensified pulse rotations buildup pea rhizosphere pathogens in cereal and pulse based cropping systems

Yining Niu, Luke D. Bainard, Zakir Hossain, William E. May, Chantal Hamel, Yantai Gan*

*** Correspondence:** [yantai.gan@agr.gc.ca](mailto:yantai.gan@agr.gc.ca)

Table S7. Spearman correlations between the assessed soil physicochemical properties or pea grain yield with soil physicochemical properties in 2016.

|  | pH | EC | Fe | Mn | Cu | Zn | PO_4_-P | K | Mg | Ca | NO_3_-N | TN | OC | TC | Moisture |
| --- | --- | --- | --- | --- | --- | --- | --- | --- | --- | --- | --- | --- | --- | --- | --- |
| EC | 0.61** |  |  |  |  |  |  |  |  |  |  |  |  |  |  |
| Fe | -0.81*** | -0.59** |  |  |  |  |  |  |  |  |  |  |  |  |  |
| Mn | -0.81*** | -0.55** | 0.92*** |  |  |  |  |  |  |  |  |  |  |  |  |
| Cu | -0.50* | -0.40 | 0.34 | 0.26 |  |  |  |  |  |  |  |  |  |  |  |
| Zn | -0.61** | -0.48* | 0.81*** | 0.87*** | 0.06 |  |  |  |  |  |  |  |  |  |  |
| PO_4_-P | 0.26 | 0.18 | -0.17 | -0.04 | -0.22 | 0.08 |  |  |  |  |  |  |  |  |  |
| K | -0.31 | -0.15 | 0.46* | 0.60** | -0.27 | 0.72*** | 0.34 |  |  |  |  |  |  |  |  |
| Mg | 0.04 | 0.35 | 0.05 | -0.05 | 0.10 | -0.05 | -0.01 | 0.04 |  |  |  |  |  |  |  |
| Ca | 0.69*** | 0.45* | -0.62** | -0.64*** | -0.14 | -0.72*** | 0.24 | -0.51* | 0.16 |  |  |  |  |  |  |
| NO_3_-N | 0.39 | 0.39 | -0.33 | -0.20 | -0.34 | -0.07 | 0.90*** | 0.35 | 0.03 | 0.30 |  |  |  |  |  |
| TN | -0.17 | -0.19 | 0.44* | 0.53** | -0.20 | 0.57** | 0.36 | 0.63** | 0.28 | -0.20 | 0.24 |  |  |  |  |
| OC | -0.31 | -0.23 | 0.42* | 0.56** | -0.25 | 0.61** | 0.16 | 0.62** | 0.12 | -0.26 | 0.02 | 0.73*** |  |  |  |
| TC | 0.03 | -0.08 | 0.26 | 0.35 | -0.22 | 0.45* | 0.43* | 0.53** | 0.31 | -0.01 | 0.34 | 0.96*** | 0.66*** |  |  |
| Moisture | -0.30 | -0.37 | 0.49* | 0.52** | -0.12 | 0.64*** | 0.18 | 0.54** | 0.29 | -0.21 | 0.04 | 0.68*** | 0.75*** | 0.63*** |  |
| Grain yield | -0.25 | -0.18 | -0.01 | 0.22 | 0.01 | -0.02 | -0.11 | -0.02 | -0.42* | -0.16 | -0.15 | -0.08 | 0.19 | -0.19 | -0.13 |

^*^ EC, Electronic Conductivity; TN, Total Nitrogen; OC, Organic Carbon; TC, Total Carbon;

^*^Values followed with an * indicates a significant correlation between the assessed soil physicochemical properties at **P* < 0.05, ***P* < 0.01 and ****P* < 0.001, *N* = 24.
