# Supplementary material for: Intensified Pulse Rotations Buildup Pea Rhizosphere Pathogens in Cereal and Pulse Based Cropping Systems
Source: Front Microbiol. 2018 Aug 23;9:1909. doi: 10.3389/fmicb.2018.01909 (PMC6115495; doi:10.3389/fmicb.2018.01909)
Supplement: Supplementary file 8 [file Table_8.DOCX]

Supplementary Material

Intensified pulse rotations buildup pea rhizosphere pathogens in cereal and pulse based cropping systems

Yining Niu, Luke D. Bainard, Zakir Hossain, William E. May, Chantal Hamel, Yantai Gan*

*** Correspondence:** [yantai.gan@agr.gc.ca](mailto:yantai.gan@agr.gc.ca)

Table S8. Significant Spearman correlations between the OTUs (> 0.2% of total reads) with assessed soil physicochemical properties in 2015.

| OTUs | pH | EC | Fe | Mn | Cu | Zn | PO_4_-P | K | Mg | Ca | NO_3_-N | TN | OC | TC | Moisture |
| --- | --- | --- | --- | --- | --- | --- | --- | --- | --- | --- | --- | --- | --- | --- | --- |
| Otu1 (*Humicola nigrescens*) | -0.15 | -0.31 | 0.17 | 0.17 | 0.21 | 0.24 | 0.08 | 0.36 | -0.11 | -0.16 | 0.07 | 0.09 | 0.3 | 0.38 | 0.46* |
| Otu11 (*Olpidium virulentus*) | -0.41* | -0.22 | 0.31 | 0.41* | 0.09 | 0.37 | 0.62** | 0.07 | -0.01 | -0.17 | 0.25 | 0.23 | 0.23 | 0.23 | -0.02 |
| Otu10 (*Penicillium levitum*) | -0.73*** | 0.04 | 0.66*** | 0.65*** | 0.49* | 0.56** | 0.47* | 0.18 | -0.35 | -0.41* | -0.06 | 0.2 | 0.38 | 0.2 | 0.02 |
| Otu4 (*Plectosphaerella cucumerina )* | 0.34 | 0.1 | -0.3 | -0.29 | -0.28 | -0.18 | 0.03 | -0.01 | 0.42* | 0.41* | 0.12 | 0.17 | -0.05 | 0.24 | 0.08 |
| Otu7 (*Fusarium redolens*) | -0.64*** | 0.09 | 0.54** | 0.58** | 0.19 | 0.46* | 0.32 | -0.01 | -0.27 | -0.38 | -0.23 | -0.09 | 0.19 | -0.06 | -0.27 |
| Otu24 (*Oidiodendron cereale*) | -0.53** | 0.04 | 0.5* | 0.48* | 0.35 | 0.37 | 0.01 | 0.05 | -0.32 | -0.38 | -0.31 | -0.17 | 0.13 | -0.14 | 0.08 |
| Otu22 (*Fusarium solani* ) | 0.36 | 0.06 | -0.36 | -0.42* | -0.27 | -0.29 | -0.31 | -0.05 | -0.02 | 0.24 | -0.4* | -0.09 | -0.21 | -0.25 | -0.23 |
| Otu12 (*Alternaria eichhorniae*) | 0.52** | 0.29 | -0.55** | -0.58** | -0.41* | -0.43* | -0.25 | -0.22 | 0.22 | 0.51* | 0.05 | 0.03 | -0.08 | -0.01 | 0.03 |
| Otu16 (*Fusarium solani*) | 0.33 | 0.32 | -0.26 | -0.35 | 0.06 | -0.34 | -0.42* | -0.02 | 0.12 | 0.22 | -0.06 | -0.13 | -0.02 | -0.06 | 0.14 |
| Otu13 (*Nectria ramulariae*) | -0.4* | -0.22 | 0.36 | 0.41* | -0.07 | 0.4 | 0.33 | 0.05 | -0.19 | -0.25 | -0.3 | 0 | 0.17 | 0.13 | -0.03 |
| Otu62 (*Chaetomium mareoticum*) | -0.6** | 0.08 | 0.54** | 0.57** | 0.39 | 0.37 | 0.22 | 0.11 | -0.15 | -0.38 | -0.06 | -0.03 | 0.39 | 0.03 | -0.12 |
| Otu2538 (*Fusarium* sp.) | -0.21 | 0.27 | 0.17 | 0.16 | -0.02 | 0.06 | -0.08 | -0.18 | -0.22 | -0.1 | -0.5* | -0.39 | -0.07 | -0.44* | -0.36 |
| Otu9 (*Fusarium graminearum*) | 0.16 | 0.21 | -0.28 | -0.24 | -0.21 | -0.25 | 0.04 | -0.22 | 0.44* | 0.39 | -0.04 | 0.26 | 0.1 | 0.17 | 0.47* |
| Otu43 (*Alternaria metachromatica*) | 0.43* | -0.14 | -0.42* | -0.48* | -0.29 | -0.28 | -0.18 | -0.09 | -0.03 | 0.37 | -0.43* | -0.07 | -0.11 | -0.09 | 0.12 |
| Otu18 (*Fusarium* sp.) | 0.30 | -0.20 | -0.28 | -0.24 | -0.04 | -0.11 | 0.16 | -0.14 | 0.22 | 0.31 | 0.43* | 0.10 | -0.05 | 0.23 | 0.12 |
| Otu42 (*Stachybotrys xanthohalonata*) | 0.51* | 0.27 | -0.54** | -0.57** | -0.32 | -0.39 | -0.3 | -0.41* | 0.45* | 0.7*** | 0.06 | 0.06 | -0.1 | 0.07 | 0.55** |
| Otu28 (*Sarocladium strictum*) | -0.03 | -0.26 | 0.16 | 0.09 | 0.4* | 0.03 | -0.01 | 0.08 | -0.16 | -0.1 | 0.04 | 0.27 | 0.18 | 0.24 | 0.22 |
| Otu35 (*Pseudapiospora corni*) | -0.71*** | -0.01 | 0.69*** | 0.66*** | 0.48* | 0.45* | 0.08 | 0.32 | -0.52** | -0.59** | -0.43* | -0.16 | 0.28 | -0.12 | -0.3 |
| Otu38 (*Dendryphion* sp.) | -0.42* | -0.45* | 0.53** | 0.46* | 0.47* | 0.42* | 0.13 | 0.43* | -0.53** | -0.51* | 0.06 | -0.01 | 0.01 | 0.11 | -0.17 |
| Otu15 (*Fusarium merismoides*) | 0.17 | -0.42* | -0.08 | -0.02 | -0.21 | 0.12 | 0.18 | 0.29 | -0.17 | -0.17 | 0.32 | -0.03 | 0.04 | 0.08 | -0.12 |
| Otu14 (*Penicillium levitum*) | -0.47* | 0.04 | 0.42* | 0.42* | 0.38 | 0.26 | 0.33 | 0.02 | -0.4* | -0.25 | -0.23 | -0.2 | 0.12 | -0.27 | -0.49* |
| Otu47 (*Subulicystidium perlongisporum*) | 0.46* | -0.08 | -0.52** | -0.49* | -0.33 | -0.5* | -0.24 | -0.3 | 0.21 | 0.43* | -0.19 | -0.36 | -0.77*** | -0.39 | -0.24 |
| Otu31 (*Fusarium* sp.) | 0.61** | 0.14 | -0.6** | -0.67*** | -0.33 | -0.48* | -0.43* | -0.24 | 0.11 | 0.61** | -0.06 | -0.41* | -0.38 | -0.32 | 0.04 |
| Otu32 (*Tetracladium* sp.) | -0.17 | -0.34 | 0.13 | 0.14 | 0.1 | 0.15 | -0.01 | 0.04 | -0.53** | -0.18 | -0.23 | -0.29 | -0.16 | -0.17 | -0.53** |
| Otu36 (*Mortierella* sp.) | 0.03 | -0.28 | -0.02 | -0.02 | -0.06 | -0.02 | -0.09 | 0.17 | -0.38 | -0.21 | -0.46* | -0.11 | -0.02 | -0.11 | -0.37 |
| Otu41 (*Chaetomium murorum*) | 0.42* | 0.01 | -0.24 | -0.35 | 0.04 | -0.38* | -0.42* | -0.36 | -0.1 | 0.19 | 0.2 | -0.49* | -0.4* | -0.54** | -0.2 |
| Otu239 (*Scutellinia* sp.) | 0.47* | 0.33 | -0.4* | -0.49* | -0.11 | -0.49* | -0.45* | -0.35 | 0.42* | 0.4* | 0.07 | 0.14 | -0.03 | 0.1 | 0.36 |

^*^ EC, Electronic Conductivity; TN, Total Nitrogen; OC, Organic Carbon; TC, Total Carbon;

^*^Values followed with an * indicates a significant correlation between OTUs and the assessed soil physicochemical properties at **P* < 0.05, ***P* < 0.01 and ****P* < 0.001, *N* = 24.
